# Infant selective attention to native and non-native audiovisual speech

**DOI:** 10.1038/s41598-022-19704-5

**Published:** 2022-09-22

**Authors:** Kelly C. Roth, Kenna R. H. Clayton, Greg D. Reynolds

**Affiliations:** 1 Developmental Cognitive Neuroscience Laboratory, Department of Psychology, University of Tennessee, Knoxville, TN 37996 USA; 2 Data Scientist at 84.51°, Cincinnati, OH 45202 USA

**Keywords:** Human behaviour, Attention

## Abstract

The current study utilized eye-tracking to investigate the effects of intersensory redundancy and language on infant visual attention and detection of a change in prosody in audiovisual speech. Twelve-month-old monolingual English-learning infants viewed either synchronous (redundant) or asynchronous (non-redundant) presentations of a woman speaking in native or non-native speech. Halfway through each trial, the speaker changed prosody from infant-directed speech (IDS) to adult-directed speech (ADS) or vice versa. Infants focused more on the mouth of the speaker on IDS trials compared to ADS trials regardless of language or intersensory redundancy. Additionally, infants demonstrated greater detection of prosody changes from IDS speech to ADS speech in native speech. Planned comparisons indicated that infants detected prosody changes across a broader range of conditions during redundant stimulus presentations. These findings shed light on the influence of language and prosody on infant attention and highlight the complexity of audiovisual speech processing in infancy.

## Introduction

Young infants experience a wide range of perceptual events, from exposure to basic sounds and sights to social interactions that increase in complexity with age and social development. How do infants find a way to process and make sense of the enormous amount of perceptual information provided by the world around them? Infants are born with basic auditory and visual processing capabilities and are able to bind relevant sounds and sights together^1–5^. These rudimentary audiovisual processing skills are further refined with experience in the natural world^6–8^. Infants improve their ability to differentiate and pick up perceptual information provided by faces and voices with increased exposure to audiovisual speech^9–13^. This improvement in multisensory processing of familiar types of faces and voices often encountered in the infant’s native environment is related to a developmental process known as perceptual narrowing^6,7,14–16^.

Perceptual narrowing occurs when perceptual sensitivity moves from being broadly tuned to a wide array of stimuli in early infancy to being more narrowly focused on relevant information routinely encountered in the infant’s native environment. The process of gaining sensitivity to native stimuli is paralleled by a loss of sensitivity to non-native or infrequently encountered stimuli. The ability to focus selective attention on functionally significant characteristics of native stimuli is critically important for the formation of perceptual expertise and recognition memory^7,17–23^. For example, by 3 months of age, infants prefer human speech over non-human speech, and if raised in a monolingual home, infants begin to show preferences for their native speech and lose perceptual sensitivity to non-native languages^24–27^. At 4 months of age, infants can discriminate vowel sounds in both their native and non-native languages. However, by 10 months of age, infants are only able to discriminate vowels in their native language if raised in a monolingual environment^28^. This heightened sensitivity results in an increased expertise in their native language at the cost of a decline in perceptual sensitivity to non-native languages.

Intersensory redundancy is another factor that affects perceptual processing in infancy. The intersensory redundancy hypothesis states that redundant information conveyed across two or more sensory modalities facilitates infant selective attention to amodal properties of multimodal stimuli^1,29–31^. Amodal stimulus properties include any information that can be processed in more than one sensory modality, allowing for information to be perceived redundantly across multiple senses. Tempo can be perceived through both the visual and auditory sensory modalities, and thus provides a good example of amodal information. In contrast, modality-specific information, such as color or pitch, can only be perceived through a single modality.

Although even newborns are capable of cross-modal matching of amodal properties of stimuli under unimodal presentation conditions^32,33^, intersensory redundancy has been shown to facilitate perceptual processing of amodal information^1,31,34,35^. Multimodal stimuli are also highly salient and infants display longer look duration to multimodal over unimodal events^36^. The temporal synchrony and shared rhythm of audiovisual stimuli recruits their selective attention to amodal properties that serve to bind auditory and visual components of the multimodal stimuli. With further development, older infants and children can process amodal information presented in unimodal events and are less dependent upon intersensory redundancy^37^. However, if a task is relatively difficult, older children will rely on intersensory redundancy to attend to and process redundant amodal information prior to processing modality-specific information of complex stimuli^38^.

Prosody, which refers to patterns of stress and intonation in speech, is a form of amodal information as it can be conveyed both through the tone of voice and facial expressions. ADS (adult-directed speech) and IDS (infant-directed speech) vary greatly in level of prosody. ADS is characterized by a relatively lower pitch, more neutral affect, and monotone inflection typical of speech used when communicating with older children and adults. In contrast, a higher pitch and more exaggerated tone variation characterizes IDS. Infants prefer the amplified pitch contour of IDS over ADS, and they show no preference for the amplitude correlated with loudness or duration patterns of IDS when compared to ADS^39^. Studies have shown infants’ attention and language-related learning is enhanced by IDS when compared to ADS^40–43^. IDS may recruit attention to redundant information by drawing infants’ gaze to the exaggerated mouth movements and tone of voice of the speaker. When attending to the mouth, infants are able to benefit from intersensory redundancy when processing amodal information across both visual and auditory modalities^44,45^. Thus, changes in speech from ADS to IDS or vice versa should be detectable if an infant is processing the amodal property of prosody.

To summarize, perceptual narrowing can result in infants gaining perceptual processing skills in their native language and losing sensitivity to non-native languages. Intersensory redundancy can help bootstrap infant selective attention and perceptual processing of amodal information such as variations in prosody associated with infant-directed and adult-directed speech. Lewkowicz and Hansen-Tift investigated the development of attention to native and non-native speech. Monolingual English-learning 4- to 12-month-old infants and English-speaking adults were shown clips of either an English speaker or a Spanish speaker using either ADS or IDS^46^. Eye-tracking was utilized to examine participants’ visual scanning patterns of the speakers’ faces. At 4 and 6 months of age, infants focused on the eyes of the speakers. Around 8 months, infants began to focus on the mouths of speakers regardless if the actor was talking in English or Spanish. The authors proposed this shift coincides with an early stage of speech development and focusing on the mouth in this stage allows infants to detect visual information that fosters word pronunciation. At 6 months of age, infants have begun to develop greater attentional control^22,47–49^ and may direct their attention to the redundant information provided by the mouth during early development of speech and language perception. Similar to adults, by 12 months of age, infants shift their attention to the eyes of the speakers when listening to native speech^46^. It is hypothesized the eye area provides additional social information to supplement speech, and this shift in attention demonstrates ongoing development of social processing skills^50–52^. However, it is expected monolingual English-learning infants would lack the same level of expertise for Spanish due to perceptual narrowing. This may explain why 12-month-old infants in non-native speech trials continued to focus on the mouths of the speaker to facilitate processing of the unfamiliar language^46^. Alternatively, it should be noted that other studies have proposed this shift in attention to the mouth could be a sign of more advanced language processing^53,54^, and studies that include vocabulary size as a factor indicate significant individual differences in the timing of the shift to and from the mouth^55,56^.

Intersensory redundancy in audiovisual speech provides a hierarchy of perceptual cues. The basic level of temporal synchrony relates to the onset and offset of the audio and visual streams. The intermediate level of temporal dynamics includes factors such as tempo, prosody, and visual synchrony. The highest level of categorical synchrony involves perception of the speaker’s affect or gender remaining congruent across audio and visual information^7,57^. The intersensory redundancy of audiovisual events, at the lowest perceptual level, is dependent on temporal synchrony between the auditory and visual amodal properties of stimuli. This lowest form of intersensory redundancy has been shown to affect infant attention during native and non-native audiovisual speech at certain ages, possibly due to perceptual narrowing. A study by de Boisferon, Tift, Minar, and Lewkowicz (2017) investigated looking behaviors of 4-, 6-, 8-, 10-, and 12-month-olds while viewing native and non-native speech presented with an audiovisual offset of 666 milliseconds^57^. The offset was determined based on prior studies that show 666 ms is sufficient for infants as young as 4 months of age to detect stimulus onset asynchrony^58,59^. The stimuli used were video clips of a native English speaker and a native Spanish speaker reciting a monologue. By comparing their findings to a previous study using synchronous audiovisual speech^46^, the authors were able to investigate how infant attention differed based on changes at the basic level of perceptual cues. Under asynchronous conditions, only the 10-month-old participants showed a significant difference in facial scanning when compared to synchronous stimuli and were the only age group to show evidence of detecting a change in basic perceptual cues^57^. Results showed 12-month-old infants looked significantly more at the mouths of the non-native (Spanish) speakers and looked at both the eyes and mouth of the native (English) speaker, but their looking patterns did not significantly differ from the synchronous results^46^.

These studies suggest that by 12 months of age infants are no longer affected by synchrony determined at the basic level (i.e., stimulus onset synchrony) in the perceptual cue hierarchy^46,57^. To our knowledge, no studies have investigated whether intersensory redundancy affects 12-month-old infants’ responsiveness to intermediate-level perceptual cues, such as prosody changes, in native and non-native speech. The current study sought to shed light on the role of intersensory redundancy in detecting intermediate-level perceptual cues in the form of changes in prosody in native and non-native audiovisual speech when provided temporally synchronous auditory and visual information. Specifically, the current study tested the effects of redundancy and language on 12-month-old monolingual infants’ responsiveness to prosody changes. At the basic level, stimulus onset and offset synchrony of audiovisual cues was consistent across both experiments. However, prosody, an intermediate-level perceptual cue conveyed within the internal elements of audiovisual stimuli, changed midway through each trial. Infants were tested to see if they would detect these changes in prosody depending on if they were presented in native or non-native speech under redundant or non-redundant presentation conditions. We predicted that non-native speech would be more difficult to process for monolingual infants at this age, but the addition of intersensory redundancy may facilitate their attention to amodal properties of speech.

The first major aim of this study focused on answering how intersensory redundancy, prosody, and language familiarity may impact the distribution of 12-month-old infants' selective attention to a speaker's facial features. The second major aim of this study analyzed infant visual recovery of looking, a marker for novelty detection, to determine if redundancy facilitates detection of a change in prosody, an amodal intermediate-level property, in native and non-native speech. Twelve months is an age when infants’ attention is less focused on processing universal audiovisual information and instead shifts to differentiating modality-specific information provided by multimodal events—making shifts to the mouths of speakers indicative of the increased task difficulty and a reliance on redundancy^60^. We were particularly interested in whether monolingual English-learning infants would notice an intermediate-level perceptual cue change (i.e., a change in prosody) in Spanish presented redundantly across the auditory and visual modalities. These two aims are labeled respectively as Selective Attention to Facial Areas of Interest (AOIs) and Prosody Discrimination.

## Predictions

### Selective attention to facial AOIs

For redundant (synchronous audiovisual) presentation conditions, we predicted monolingual English-learning 12-month-old infants would focus equally on the eyes and mouth of the native speaker during redundant (synchronous) trials^46^. By 12 months of age, English-learning infants should be highly familiar with their native speech and not need to rely on the mouth as much for redundant information as in earlier development^61^. Non-native Spanish trials should also be more difficult to process for infants due to lack of exposure, which should lead infants to focus on the mouth of the non-native speaker^46^.

For non-redundant (asynchronous audiovisual) presentation conditions, we assumed non-redundant native English video clips would be more difficult than redundant clips for 12-month-old infants to process. We predicted this may lead infants to rely on intersensory redundancy to process the stimuli, shifting their gaze proportionately more to the mouth of the native speaker in comparison to redundant trials^61^. Non-native Spanish trials should also be more difficult to process for infants due to lack of exposure to the language, which would lead infants to focus more on the mouth of the Spanish speaker regardless of redundancy^44,46^.

### Prosody discrimination

For the redundant presentation condition, we predicted infants would demonstrate visual recovery based on changes in prosody for both native and non-native speech^62^. We hypothesized intersensory redundancy would enable infants to detect amodal intermediate-level changes, such as when the speaker changes from one prosody to the other. Since intersensory redundancy directs infant selective attention to, and facilitates perceptual processing of, amodal properties of audiovisual speech, we predicted that infants would be able to detect the prosody change, even in non-native speech^1^.

For the non-redundant presentation condition, we predicted infants would only demonstrate visual recovery based on an intermediate-level cue change in prosody during native speech, but not non-native speech^45^. We hypothesized infants’ familiarity with their native language would facilitate their detection of a change in prosody, regardless of the lack of intersensory redundancy^37^. However, due to the lack of intersensory redundancy bootstrapping attention to the prosody conveyed in speech, we predicted infants would not notice the change in prosody in the more difficult non-native speech^60^.

## Method

### Participants

The final dataset included 40 monolingual English-learning infants (39 White, 1 Black; 25 male, 15 female) with a mean age of 365.55 days (*SD* = 6.49, *range* = 356–378). Participants were all born full-term (no less than 37 weeks gestation) without complications during pregnancy or delivery and had no known visual difficulties or other major health issues. Participants were recruited without regard to race, ethnicity, or gender.

Infants with significant exposure to a language other than English, more than an occasional encounter as reported by their caregivers, were excluded. This included infants who had a bilingual parent or caretaker or whose parents intentionally exposed the infant to a non-native language as part of learning enrichment. An additional 40 infants were tested but excluded from these analyses due to significant experience with Spanish or a language other than English (*N* = 4), inability to validate eye-tracker calibration due to technical difficulties (*N* = 12), fussiness (*N* = 5), or inadequate number of useable trials (*N* = 19).

### Apparatus

Infants were seated in their parent’s or guardian’s lap throughout testing. Participants were seated approximately 60 cm away from a 17″ Dell UltraSharp 1704FPV color LCD monitor, which displayed the stimuli for testing. There were Dell desktop computer speakers positioned behind black fabric surrounding the monitor to provide sound presented at 55 dB during audiovisual trials without being a visible distraction. Testing took place in a sound-attenuated room that was darkened with light-blocking shades and black curtains. Stimuli were presented through Experiment Builder software (SR Research Ltd., Mississauga, Ontario, Canada) on a Dell Windows PC. A remote eye-tracker (SR Research Ltd., Mississauga, Ontario, Canada, Eye-Link 1000 Plus) was positioned under the LCD monitor, facing the infant, to record eye scanning patterns with a 500 Hz sampling rate. The calibration of the eye-tracker was performed through a three-point calibration procedure. Animated cartoon characters moving and sounding in concert appeared pseudo-randomly at the top center, bottom-left, and bottom-right of the LCD monitor. Calibration was repeated until the infant attended to all three points, and a validation procedure of the same points confirmed adequate calibration accuracy.

### Visual stimuli

The stimuli were 30 s audiovisual video clips of a woman, either a native English speaker or native Spanish speaker, talking in either IDS or ADS, followed by an immediate switch to the other prosody for an additional 30 s. Auditory analysis of the average, maximum, and minimum of the fundamental frequency (f0) of the stimuli are as follows: English ADS average f0: 209.30 Hz min: 85.53 Hz max: 348.00 Hz; English IDS average f0: 233.38 Hz min: 85.38 Hz max: 524.90 Hz; Spanish ADS average f0: 247.1 Hz min: 80.06 Hz max: 485.45 Hz; Spanish IDS average f0: 292.60 Hz min: 113.58 Hz max: 570.25 Hz. The stimuli were provided by and used with permission from Dr. David Lewkowicz, and the actors portrayed in the videos provided consent to publish their personal images in publications^46^. Order of presentation for prosody and language was counterbalanced across participants to control for potential order effects. Both the native and the non-native speaker had their hair pulled back from their face and were filmed against a plain black background. The actors delivered their monologues while standing still and there were no hand gestures or overt movements. Only their facial expressions and tone of voice changed between prosodies.

Half of the participants (N = 20) were tested in the redundant (synchronous audiovisual) testing condition and half (N = 20) were tested in the non-redundant (asynchronous audiovisual) testing condition. Redundancy refers to the temporal synchrony of the video clips of the women speaking. In the redundant condition, the audio and video components were synchronous (thus providing intersensory redundancy), and the video played normally with matching video and audio tracks. In the non-redundant condition, the video clips were the same clips used in the redundant condition. However, the audio was edited to not play in synchrony with the video, although both the audio track and the video onset occurred at the same time. This preserved the basic-level perceptual cues of stimulus onset and offset synchrony but disrupted the internal temporal synchrony of the movements of speech with respect to the temporal structure of the speech sounds. Thus, although prosody was conveyed in both the visual and auditory components of the stimuli, the intersensory redundancy for the intermediate-level prosodic cues was disrupted by the lack of synchrony. These asynchronous, non-redundant videos were created using the video editing software iMovie (version 10.1.8. Apple Inc. 2017. Mac OS X 10.12.6). The audio track was divided into two halves, then played in the opposite order. Infants heard the second half of the audio track followed by the first half. The first mouth movement of the speaker was synchronized with the first sound of the audio track to ensure stimulus onset synchrony. Infants in the non-redundant received the same audio and visual information as in the redundant condition but did not have the added benefit of intersensory redundancy.

### Procedure

All procedures associated with this study followed a protocol approved by the Institutional Review Board of the University of Tennessee, Knoxville and were carried out in accordance with relevant guidelines and regulations. Caregivers of the infants were asked what languages were spoken at home and for an estimate of total possible exposure to non-native speech to determine if infants qualified as monolingual English learners. The experimenter went over the procedure and obtained informed consent from the parent or legal guardian. The adult was instructed to sit holding the infant in front of the monitor and to refrain from moving or making noise during testing. Once seated, a small bullseye pattern sticker was placed on the infant’s forehead to aid the eye tracker with pupil localization for calibration and tracking scanning patterns.

**Figure 1:**
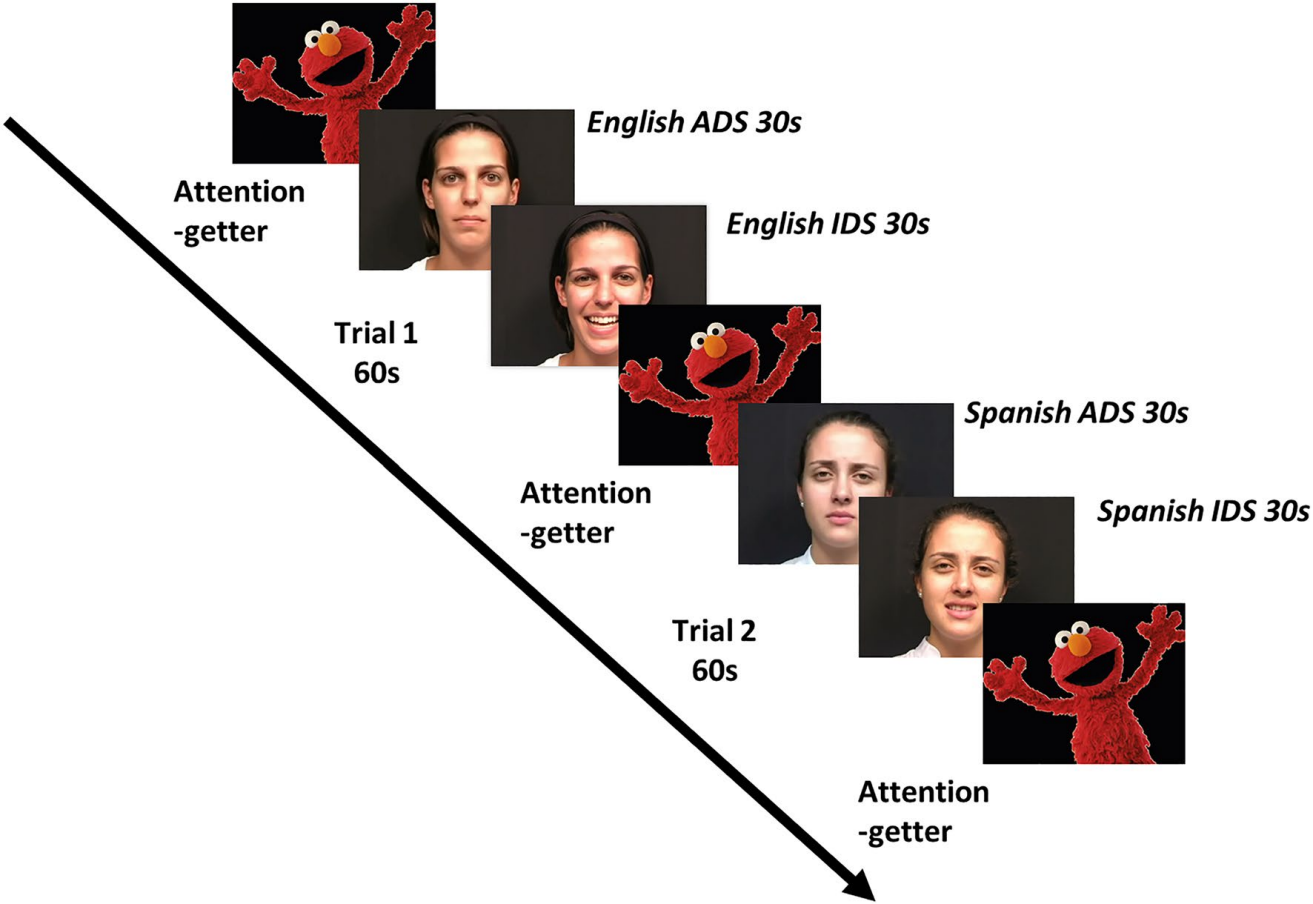
Example of first block of procedure used in Experiments 1 and 2. Stimuli credit to Lewkowicz and Hansen-Tift^46^.

After calibration, an attention-getter, an audiovisual clip of Sesame Street, was played to centrally fixate the participant. A trial consisted of a single speaker, either English or Spanish, speaking for 60 s, switching prosody (either from ADS to IDS or from IDS to ADS) at 30 s. Following the 30 s of speech after the prosody switch, an attention-getter was presented in the center of the monitor until the infant was centrally fixated. Then the second trial begin with whichever speaker the infant did not see first, playing for 60 s in the same prosody order as the first one with the prosody switch at 30 s (see Fig. 1 for example of a block of trials). The experiment had 2 blocks comprised of 2 trials each for a total of 4 trials. Infants had to be centrally fixated to the screen at the beginning of each of the 4 trials to be considered for the final dataset. Both groups of infants viewed a native (English) speaker and a non-native (Spanish) speaker during Block 1, and the same order of language (native first or non-native first) but a counter-balanced order of prosody for Block 2 (e.g., if infants heard ADS to IDS in Block 1, they heard IDS to ADS in Block 2). Order of language and prosody were counter-balanced across participants.

### Dependent variables

**Figure 2:**
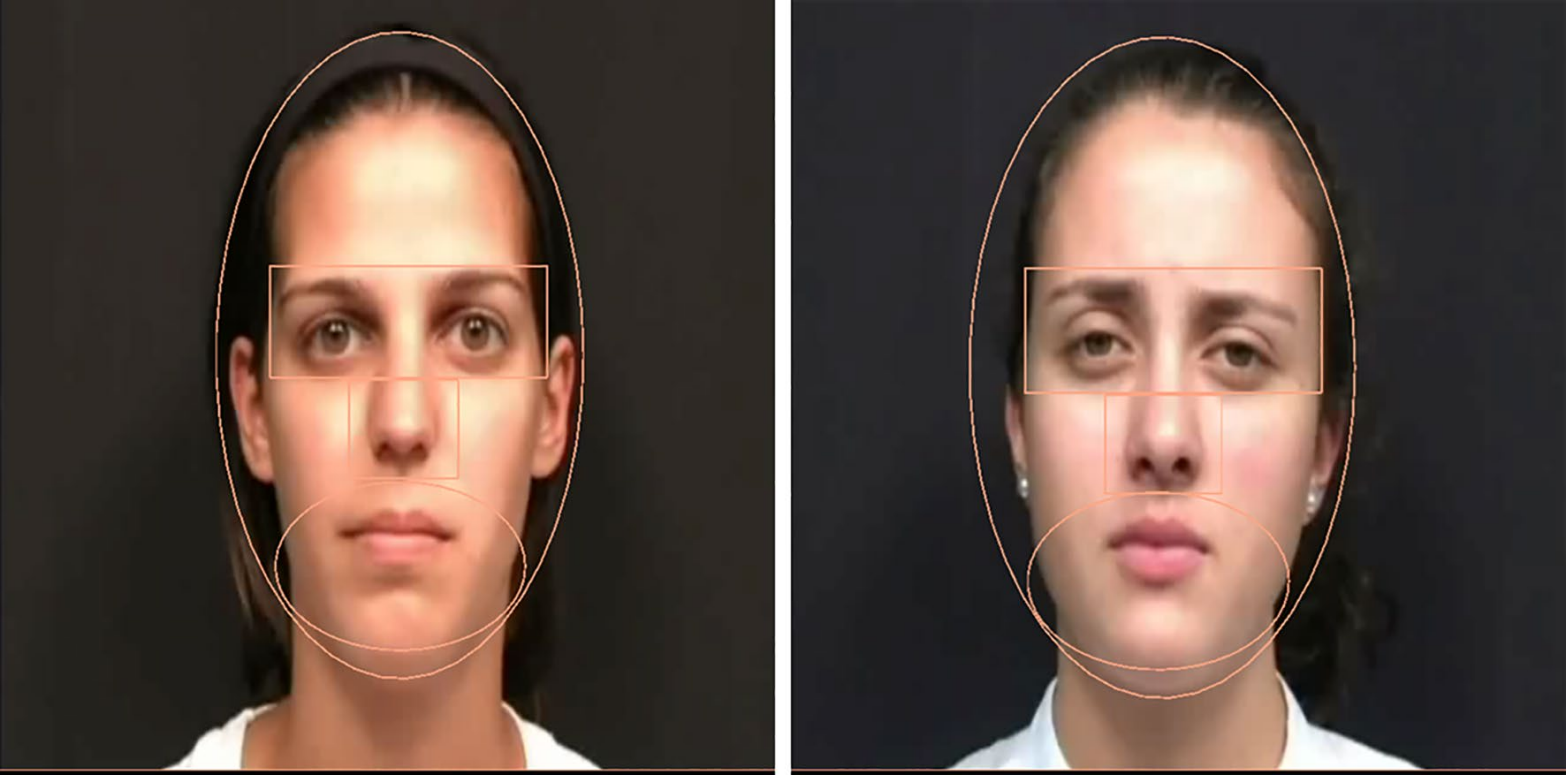
Example of stimuli used in Experiments 1 and 2 with AOIs for analysis overlaid. Left image is the English (native) speaker and right image is the Spanish (non-native) speaker. AOIs include the face, the eyes, and the mouth. Stimuli credit to Lewkowicz and Hansen-Tift^46^.

To measure selective attention to facial features, both speakers’ faces were divided into three AOIs. AOIs were defined as a horizontal rectangle around both eyes, an oval around the mouth area, and an oval around the entire face (see Fig. 2). When viewed from 55 cm, the visual angle subtended by the AOI for the eyes was 10.4° × 4.2°. The visual angle subtended by the AOI for the mouth was 9.4° × 6.2°, and the visual angle subtended by the entire face was 24.1° × 13.5°. Proportion of looking was calculated by equating the total duration of fixations on the face to 1.00 and determining what proportion of dwell time duration on the face occurred within each AOI.

To measure detection of prosody change, the difference between the sum of fixations to the screen during the final 5000 ms prior to a prosody change and the sum of fixations to the screen during the first 5000 ms following a prosody change (i.e., the prosody change that occurred after 30 s in each trial) was measured as an index of visual recovery of looking. Visual recovery of looking is evidenced by an increase in looking to a novel stimulus after habituation to a familiar stimulus and used as a marker of novelty detection^63^.

### Design for statistical analysis

Selective attention and prosody change detection data were fitted separately with a mixed effects model using the lme4 package in R^64^. The selective attention model comprised of 5 fixed effects: synchrony (redundant, non-redundant), order (ADS first, IDS first), language (English, Spanish), prosody (ADS, IDS), and AOI (eyes, mouth). To account for participant-specific differences in looking behavior, the model included participant as a random factor in addition to a random slope of AOI to account for potential individual differences in attentional shifts^55,56^. The change detection model included 4 fixed effects: synchrony, order, language, and block (ADS to IDS, IDS to ADS) and a random factor of participant. Estimated coefficients of fixed effects were evaluated using omnibus ANOVAs to determine main effects and their interactions. All *p*-values were calculated using Satterthwaite’s approximation. For significant effects, post-hoc, multiple pairwise comparisons were conducted using the *emmeans* package with Bonferroni correction. Degrees of freedom were calculated using the Kenward Roger method.

### Selective attention to facial AOIs

**Figure 3:**
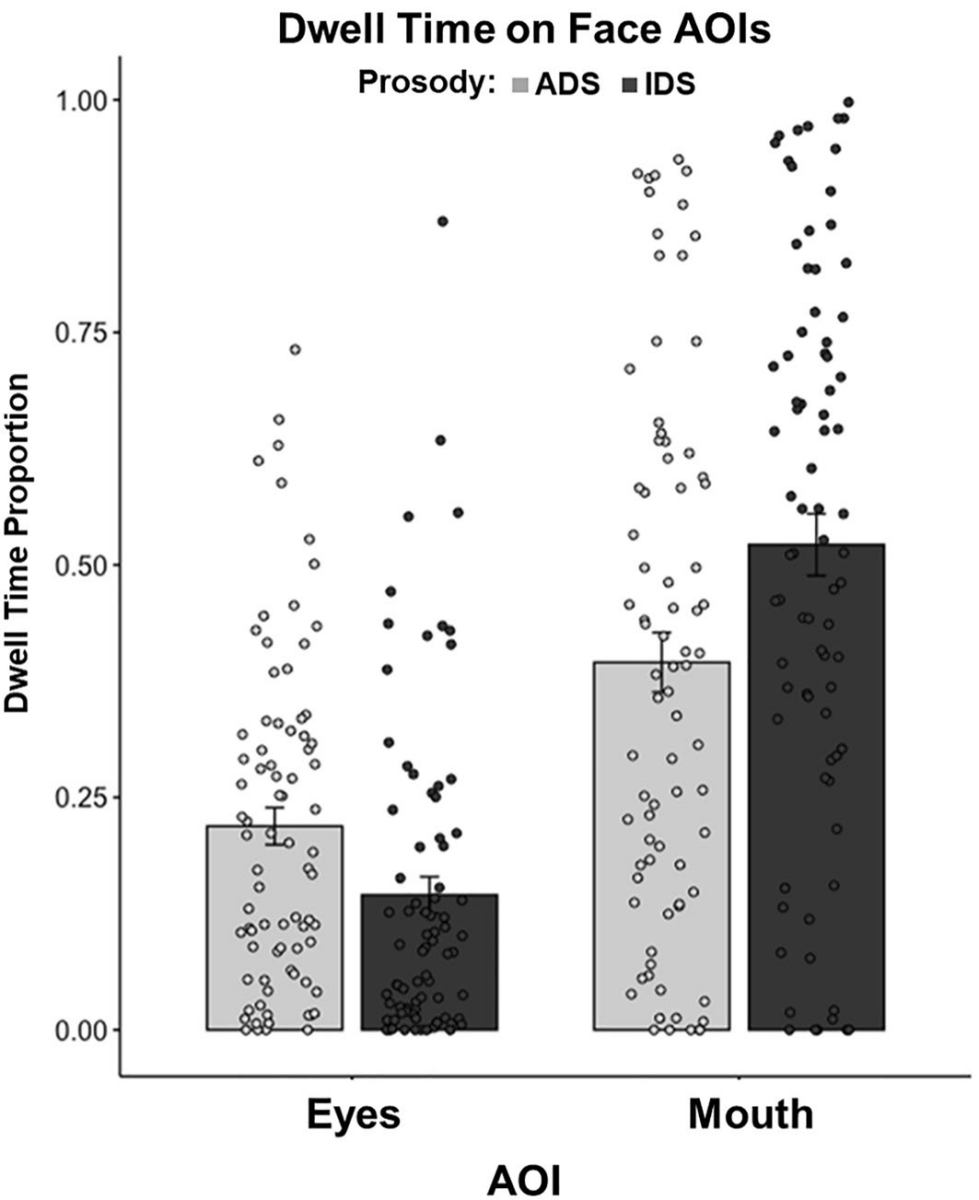
Plot of individual data on the proportion of infant dwell time, compared across AOIs and prosody (adult-directed speech or infant-directed speech). Proportion of looking was calculated by equating the total duration of fixations on the face to 1.00 and determining what proportion of dwell time duration on the face occurred within each AOI. Error bars represent standard error of the mean.

In the selective attention model, there was no main effect of synchrony found (*F*(139.7) = 0.142, *p* = 0.708). There was a significant main effect of AOI (*F*(139.9) = 20.982, *p* < 0.001) in addition to a significant two-way interaction of AOI and prosody (*F*(1240.0) = 49.577, *p* < 0.001)*.* Post-hoc, pairwise comparison shows that infants looked to the mouth (*EMM* = 0.459, *SE* = 0.042) more than the eyes (*EMM* = 0.183, *SE* = 0.024); (*t*(42.1) = 4.465, *p* < 0.001). Post-hoc, pairwise comparisons deconstructing the interaction of AOI and prosody reveal that infants looked more at the mouth during IDS (*EMM* = 0.523, *SE* = 0.043) than the mouth during ADS (*EMM* = 0.396 *SE* = 0.043); (*t*(245.1) =  − 6.218, *p* < 0.001). Conversely, infants looked more at the eyes during ADS (*EMM* = 0.220, *SE* = 0.026) than during IDS (*EMM* = 0.523, *SE* = 0.043); (*t*(245.1) = 3.636, *p* < 0.01). See Fig. 3 for plotted individual data showing AOI dwell time by prosody. Additionally, heatmaps illustrating the distribution of infant visual scanning by language and prosody have been added to Supplementary Materials for the interested reader.

### Prosody discrimination

To analyze prosody change detection, we compared change scores obtained by subtracting look duration from the last 5 s prior to a prosody change from look duration from the first 5 s following the prosody change. Positive scores represent visual recovery with an increase in looking following the prosody change. In the change detection model, there was no main effect of synchrony found (*F*(1.40) = 0.114, *p* = 0.737).

**Figure 4:**
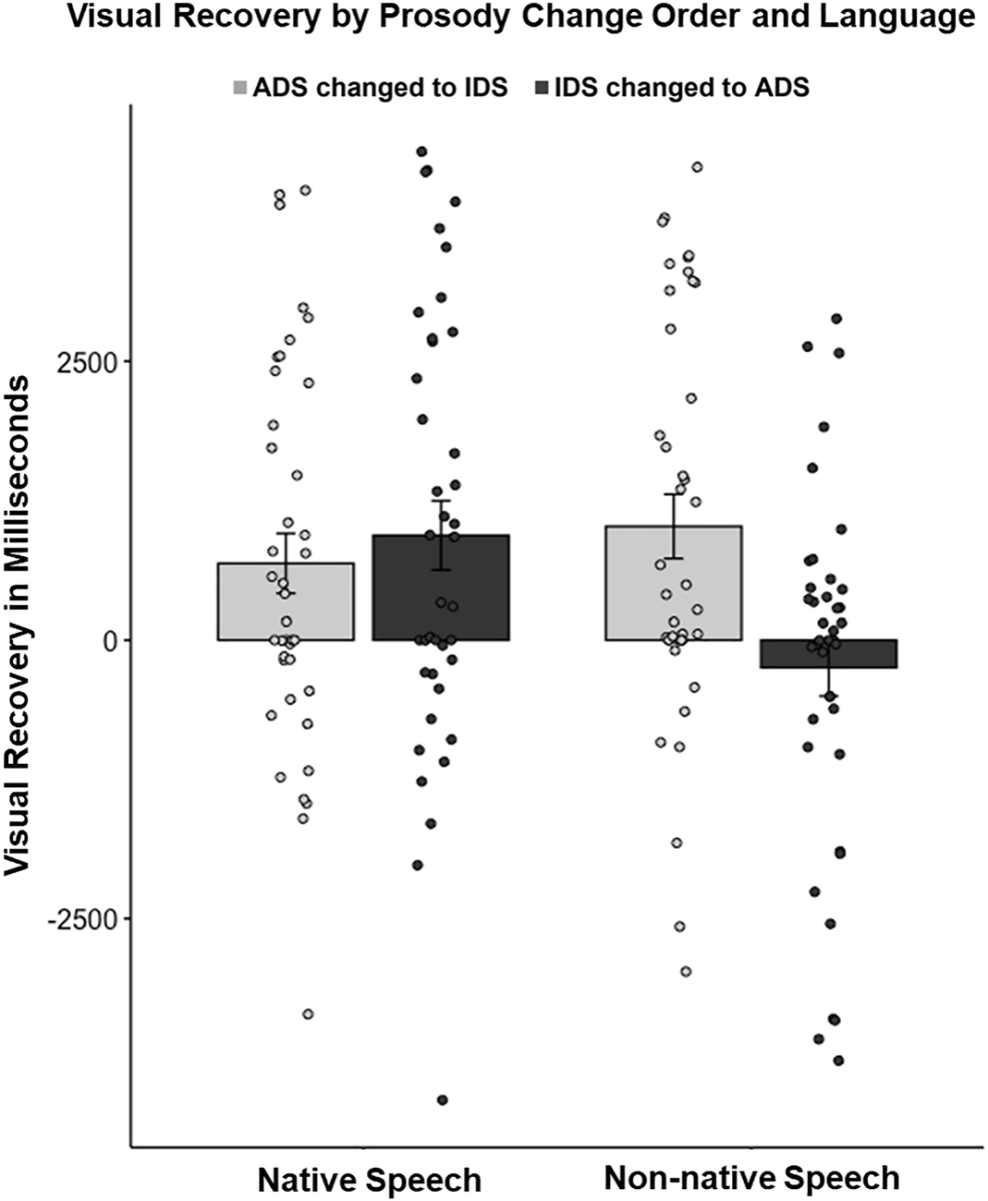
Plot of individual data of infant visual recovery in milliseconds after the prosody change, compared across language and order of prosody change. Bars represent average change scores by condition. Error bars represent standard error of the mean.

As can be seen in Fig. 4, there was a significant interaction of language and block (*F*(1120) = 8.612, *p* < 0.01). Infants detected prosody change more in IDS to ADS blocks (dark bars) in the native speech (*EMM* = 946*, SE* = 284) condition compared to non-native speech (*EMM* =  − 238, *SE* = 284); (*t*(123) = 3.20, *p* = 0.01). The IDS to ADS change in non-native speech was the only condition in which infants continued to show a decrease in looking following a prosody change.

### Prosody change detection

The analysis of prosody change presented above compared the magnitude of change scores across conditions. To examine infants’ ability to detect a change in prosody in audiovisual speech, paired samples *t*-tests comparing look duration from the first 5 s following a prosody change to look duration from the last 5 s prior to the change were used to determine whether the change in looking was significant within each experimental condition. As can be seen in Fig. 4, regardless of synchrony (redundant, non-redundant), infants demonstrated significant visual recovery when prosody shifted from ADS to IDS (*t*(39) 2.552, *p* = 0.015) and when prosody shifted from IDS to ADS (*t*(39)3.015, *p* = 0.004) in native speech, and when ADS changed to IDS in non-native speech (*t*(19) 2.351, *p* = 0.030). Infants also showed significant visual recovery when ADS changed to IDS in non-native speech (*t*(39) 3.524, *p* = 0.001). No evidence of visual recovery was found when IDS changed to ADS in non-native speech (*p* = 0.338).

**Figure 5:**
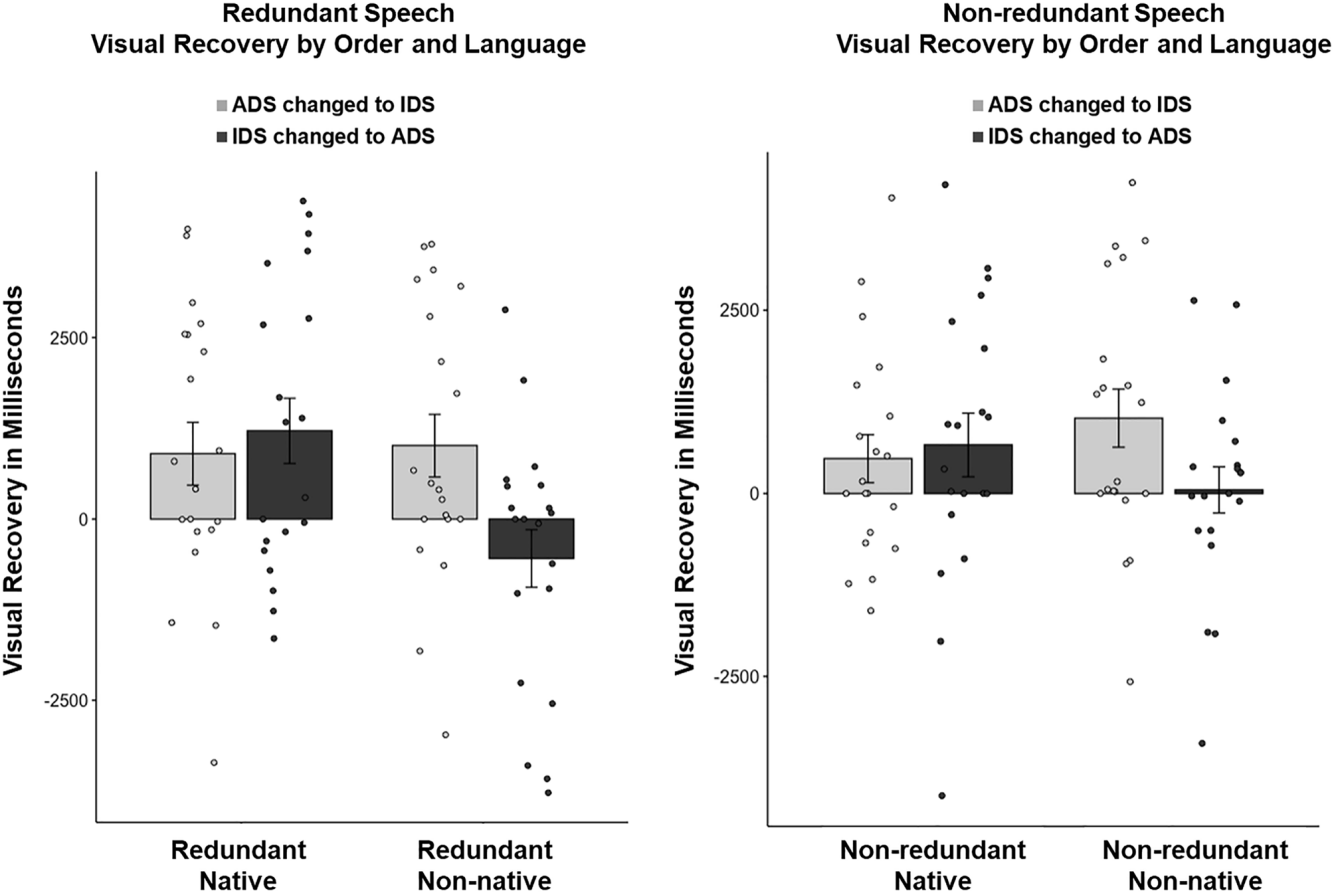
Plot of individual data of infant visual recovery in milliseconds after the prosody change, compared across language and order of prosody change. Bars represent average change scores by condition. Error bars represent standard error of the mean. The redundant condition is shown in the left panel and the non-redundant condition is shown in the right panel.

In order to test our a priori hypotheses that intersensory redundancy and language would impact prosody change detection, we performed planned comparisons separately for each synchrony condition (redundant, non-redundant). The results can be seen in Fig. 5. Infants in the redundant condition (Fig. 5, left panel) demonstrated significant visual recovery when prosody shifted from IDS to ADS in native speech (*t*(19)2.709, *p* = 0.014), and when ADS changed to IDS in non-native speech (*t*(19) 2.351, *p* = 0.030). Infants in the redundant condition showed marginally significant visual recovery when ADS changed to IDS in native speech (*t*(19) 2.085, *p* = 0.051). No difference was found in the redundant condition when IDS changed to ADS in non-native speech (*p* = 0.188).

Infants in the non-redundant condition (Fig. 5, right panel) only showed significant visual recovery when ADS changed to IDS in non-native speech (*t*(19) 2.581, *p* = 0.018, all other *p*s > 0.10).

## Discussion

Results of the current study indicate 12-month-old infants’ selective attention to facial features was affected by prosody more so than language or intersensory redundancy. We predicted non-redundant native speech would be more difficult to process than redundant native speech, therefore infants would look more at the mouth to process the amodal information being conveyed without the facilitating effects of intersensory redundancy^61^. We also predicted infants would look more at the mouth of the non-native speaker, regardless of prosody or redundancy, due to the greater difficulty of processing an unfamiliar language^44,46^. These predictions were supported by the data, as there was a main effect of AOI and infants overwhelmingly attended to the mouths of speakers across all conditions. However, we also predicted that infants in the redundant condition would shift their gaze equally between eyes and mouth for the native speaker based on previous research of this age group^46^. Yet, the current findings indicate infants focused more on the mouth than the eyes during redundant native audiovisual speech, which may be due to the use of highly salient infant-directed speech for part of each trial^65–67^.

Overall, our findings indicate that 12-month-old English learning infants look at the mouths of the speaker regardless of intersensory redundancy or language, and infants look proportionally more to the mouth of the speaker during IDS compared to ADS trials and more to the eyes during ADS compared to IDS trials. Infant selective attention to facial features may not be affected by the presence or lack of intersensory redundancy in this context, which is consistent with previous studies that have found no basic synchrony effects for 12-month-old infant visual scanning of faces (e.g., de Boisferon, Tift, Minar, & Lewkowicz’s 2017 study)^57^. These findings suggest that by 12 months of age infants may not rely as heavily on redundant amodal information given by the mouth as they do at earlier ages^14,68,69^.

In our analysis of detection of changes in prosody, the current findings revealed an interaction of language and block (i.e., the direction of change in prosody). Specifically, the magnitude of change detection was greater in native speech compared to non-native speech when IDS changed to ADS. The salience of IDS combined with familiarity with native speech likely contributes to this interaction^41,42,46,65^. The only condition in which infants did not demonstrate significant visual recovery was when IDS changed to ADS in non-native speech. It may be the case that the salience of IDS influenced infant attention to a greater extent in non-native speech, and that infant attention was influenced to a greater extent by stimulus changes in native speech. Thus, infants demonstrated recovery of looking when prosody shifted from ADS to IDS as well as from IDS to the less perceptually salient ADS in native speech, but infants only showed recovery of looking when prosody shifted from ADS to the highly salient IDS in non-native speech.

While we hypothesized intersensory redundancy would bootstrap infants’ attention to amodal properties of non-native speech, it seems it did not have the predicted effect at this age, which may be related to the ongoing development of speech and word learning in their native language^70^. Redundancy did not have a significant impact on the magnitude of change scores in our prosody change analysis. However, planned comparisons indicated intersensory redundancy does influence the conditions in which infants detect changes in prosody. Infants demonstrated significant recovery of looking across a broader range of prosody change conditions under redundant presentation conditions than under non-redundant presentation conditions. Greater familiarity with English combined with intersensory redundancy provided by synchronous audiovisual speech may boost infants’ ability to detect changes in prosody, an intermediate-level perceptual cue. Additionally, we predicted infants would be able to discriminate a change in non-redundant native speech but not in non-redundant non-native speech. However, the results were more nuanced and inconsistent with this prediction overall. Infants detected an intermediate-level change in prosody in both non-redundant native and non-native speech when prosody changed from ADS to IDS. Without the naturalistic presentation of redundant speech, both languages were presented in a novel format during the non-redundant conditions, and this may have led infants to focus exclusively on the salience of IDS.

Danielson and colleagues studied auditory phonetic discrimination of syllables in native and non-native audiovisual speech in 6- to 12-month-old infants^71^. Infants’ sensitivity to congruence between seen and heard speech in a non-native language was examined using eye-tracking, and the authors tested the possibility that familiarization with congruent or incongruent audiovisual speech could boost auditory discrimination of non-native syllables. Because research has shown that the tendency for 8- to 12-month-old infants to fixate on a speaker’s mouth is more pronounced with non-native speech^44,46^, and 6- to 9-month-olds also fixate more on the mouth when observing incongruent audiovisual speech in their native language^72^, it was predicted that familiarization with incongruent audiovisual speech would facilitate non-native syllable discrimination for older infants at an age when such discrimination is typically no longer demonstrated. Results indicated that familiarization to incongruent audiovisual speech influenced subsequent auditory discrimination at test for 6-month-olds but not for older infants.

Shaw and Bortfeld (2015) have cited current issues in infant audiovisual speech perception research, outlining how comparing data despite methodical and stimulus differences may contribute to difficulty in interpreting the results^73^, and Maempel (2019) has since made a call for standardized measurements for audiovisual perception research^74^. Lewkowicz and Bremner (2020) acknowledge this is partly due to so many of these studies being demonstration studies that do not directly correlate to one another^75^. While our findings for the redundant non-native trials do not dovetail perfectly with other research, we are aware of how different the experimental paradigms are for the referenced studies. The current experiment used a change detection task that showed stimuli that differed at the intermediate level of perceptual cues and measured infants’ recovery of looking. A similar study on 12- to 14-month-old infants used the same stimuli as the current study in a visual paired-comparison procedure. The native and non-native speakers’ videos were played on either side of the infant and the matching sound for one of the videos was played centrally. Infants were able to correctly pair the non-native speech with the non-native video when the audio and visual streams were played in synchrony^62^. Another study using a similar methodology of paired-preference trials tested German learning infants with two videos of an actor speaking German and the same actor speaking French while audio for one of the videos played. Interestingly, the researchers found that the 12-month-old infants looked significantly longer at the *non-native* speech (French) when the French audio was playing in synchrony, yet the infants did not show this audiovisual matching when their native German was played^44^. Thus, these two studies indicate that 12-month-old infants are capable of processing non-native audiovisual speech when compared to their native speech, but both studies used a visual paired-comparison procedure. In contrast, the current study used a change detection task similar to habituation measures and found the 12-month-old infants were able to detect a change in prosody in both native and non-native speech under specific change conditions.

It should be noted that we used native and non-native IDS and ADS as a metric, but studies show that the acoustic properties of IDS differ across languages^76,77^. The focus of our research was to determine if infants detect a change in prosody within either their native (English) or a non-native (Spanish) language. However, the differences in pitch contour between Spanish ADS and IDS were greater than the pitch contour differences in English ADS and IDS (45.5 Hz > 24.08 Hz), and this may have aided infants’ detection of prosody changes in Spanish. Additionally, our stimuli were not matched on emotional expression (i.e., affect). IDS contains a much more positive and exaggerated affect than ADS. Thus, it is possible infants were perceiving the difference in the higher-level of affect as opposed to the difference in prosody. Using two prosodies that were matched in emotional intensity could better measure whether infants detected a change in prosody instead of a change in emotional intensity. The perceptual salience of IDS in comparison to ADS^39^ likely had a significant impact on the current findings on prosody detection. Future studies should examine infants’ ability to detect changes in amodal properties of stimuli which may be more difficult to detect and more readily equated on factors such as affect (e.g., tempo or rhythm).

## Conclusion

Infants are born with fundamental auditory and visual processing capabilities. They are able to loosely bind these separate streams of sensory information into cohesive multisensory events based on shared basic-level perceptual cues, such as temporal synchrony or intensity^1,3^. This ability to bind audiovisual events at a basic level is broadly tuned and is not specific to native or non-native stimuli^2,26^. Infants’ experience binding basic-level auditory and visual information provides a foundation that informs more nuanced and complex audiovisual processing with experience^11^. As infants begin to build expertise in processing native stimuli, there is a corresponding loss of sensitivity to non-native stimuli, a process referred to as perceptual narrowing^6,7,14–16^. While infants initially process stimuli at the most basic level, age and experience lead to more complex processing, including discrimination of intermediate-level perceptual cues such as tempo or prosody and high-level cues such as the speaker’s affect or gender^7,57^.

The current study sought to explore how 12-month-old infants respond to perceptual changes in native and non-native speech at the intermediate level when basic-level perceptual information was held constant. Findings suggest that the salience of infant-directed speech and intersensory redundancy both play an important role in infants processing perceptual properties of audiovisual speech. Based on the current and extant findings in the area, multiple factors, such as familiarity with a given language, prosody, and direction of stimulus changes, all play a role in recruiting infant selective attention and fostering perceptual processing of audiovisual speech. Future research should investigate these factors further by manipulating a broader range of perceptual cue conditions and testing a broader range of age groups. Understanding how this network of influences affects infant selective attention across early development will provide a more comprehensive understanding of infant perceptual development and audiovisual speech processing.

## Supplementary Information

Supplementary Information.

## Data Availability

The datasets from the current study are freely available from the corresponding author upon reasonable request.
