# Supplementary material for: Infant selective attention to native and non-native audiovisual speech
Source: Sci Rep. 2022 Sep 22;12:15781. doi: 10.1038/s41598-022-19704-5 (PMC9500058; doi:10.1038/s41598-022-19704-5)
Supplement: Supplementary file 1 — Supplementary Information. [file 41598_2022_19704_MOESM1_ESM.docx]

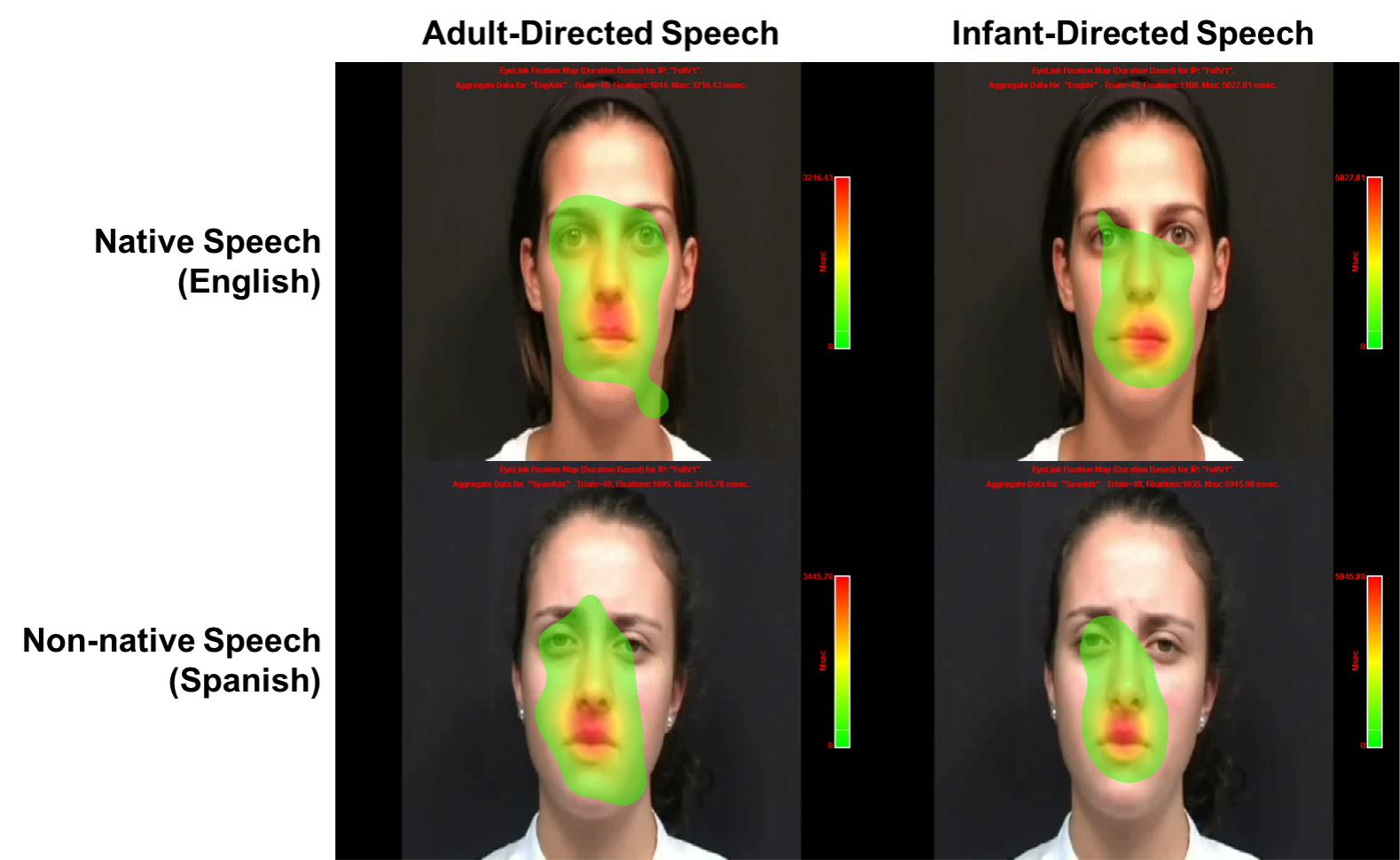


Supplemental Figure S1.

Aggregated heat maps showing distribution of infant selective attention during the 30 second pre-change trial, segmented by Language and Prosody. Starting at the top-left and moving clockwise, images depict native ADS, native IDS, non-native ADS, non-native IDS.
